# Tannate of Cannabine

**Published:** 1885-02

**Authors:** 


*Tannate of Cannabine.*—Tetanus-cannabine is a crystallized substance extracted from cannabis indica by the eminent Prof. Mathew Hay, in Scotland. It is known that this tetanus-cannabine, possessing most potent toxic properties, resembles strychina in its physiological action. It is obtained in the form of colorless crystalline needles, soluble in water, in alcohol, and much less in chloroform and ether. If we isolate one of the parts, constituting this extractive matter so far unknown, which Prof. Hay has named *tetanus-cannabine*, to remember its physiological properties and its origin without prejudicing its true nature, and if we unite to tannin the substance which contains the hypnotic properties of cannabis indica, we will obtain a new body called *tannate of cannabine*, which possesses narcotic properties by far superior to the extract of cannabis indica. Dr. Trohmüller had used it with the best results. He refers to the history of 63 patients, 21 males and 42 females, the ages ranging from 17 to 77 years, the majority being between 20 to 40 years. Of this number 40 were affected with phthisis, 4 with abdominal tumors, 3 with chronic bronchitis, 2 with lead colic, 1 with acute pneumonia, 1 with dementia, 2 with alcoholism, a female had perimetritis, 2 other patients were severely affected with asthma, 4 presented painful symptoms of mercurial poisoning, a last one had an abdominal neuralgia.

All these complained of a complete or partial insomnia, for which they had taken opiates per os, or morphine hypodermically. The tannate of cannabine gave good results as a sedative and a hypnotic in 37 of these patients. The action of the remedy began to be noticed about half an hour after its absorption by the mouth, and the sleep continued calm during all the night. In 15 other patients the calm was only partial, while the result was negative in other 12 subjects. Two patients, on awakening, complained of a slight head turbidity, which changed to headache or to a vertigo in some cases. A patient, who had taken a larger dose of the tannate of cannabine, presented symptoms of narcotism, which were relieved with acetic ether. In no case occurred any constipation nor nausea.

In the *Algens. Med. Chir. Zeitung*, Dr. Trohmüller calls attention to the advantages of this remedy to opium. It does not affect the physiological secretions, and less than opium, there occur any toxic accidents. All this will tend to make the tannate of cannabine a potent rival of opium.

The therapeutical dose is one grain (5 centigr.) to 5 grains (25 centigr.). It is important that the tannate of cannabine be free of any trace of tetanus-cannabine. (*Riv. Clin. e terap.*)

[It has no action when there exists a great excitement. It is to be absolutely avoided in case of hallucinations, for it will increase their intensity.—L.]

